# Genistein-induced mitochondrial dysfunction and FOXO3a/PUMA expression in non-small lung cancer cells

**DOI:** 10.1080/13880209.2022.2123933

**Published:** 2022-10-06

**Authors:** Liujia Chan, Yuheng Pang, Yuji Wang, Di Zhu, Ayijinag Taledaohan, Yijiang Jia, Lichun Zhao, Wenjing Wang

**Affiliations:** a School of Pharmacy, Guang Xi University of Chinese Medicine, Nanning, PR China; b Beijing Institute of Hepatology, Beijing YouAn Hospital, Capital Medical University, Beijing, PR China; c Department of breast cancer surgery, Harbin Medical University Cancer Hospital, Harbin Medical University, Harbin, PR China; d College of Pharmaceutical Sciences, Capital Medical University, Beijing, PR China

**Keywords:** Lung carcinoma, phytoestrogenic compound, mitochondrial apoptosis

## Abstract

**Context:**

Genistein is a multifunctional natural compound.

**Objective:**

In this study, we demonstrate the activity of genistein on non-small lung cancer A549 and 95D cells.

**Materials and methods:**

A CCK8 assay was used to detect the cytotoxicity of genistein (0, 25, 50, 100, 150, 200 and 250 μM) on A549 and 95D cells for 48 h. AnnexinV-FITC/PI and TUNEL assay were performed to examine the apoptotic cell death induced by genistein (0, 50, 100 and 150 μM, 48 h). Intracellular reactive oxygen species (ROS) generation and mitochondrial membrane potential were measured by flow cytometry. Mitochondrial activity in A549 and 95D cells, treated with 0, 50, 100 and 150 μM genistein for 48 h was detected by MitoTracker Orange staining. Western blot analysis was performed to evaluate the expression of the mitochondrial apoptosis-related proteins. Meanwhile, the expression level of FOXO3a/PUMA signalling was measured by flow cytometry and Western blot assay.

**Results:**

IC_50_ value of genistein against 95D cells and A549 cells was 32.96 ± 2.91 and 110.6 ± 2.41 μM, respectively. The percentage of apoptotic death cells was 20.03%, 29.26% and 27.14% in 95D cells, and 41.62%, 55.24% and 43.45% in A549 cells when treated with 50, 100 and 150 μM genistein, respectively. Our observations also revealed that genistein could elevate intracellular ROS generation, decrease mitochondrial membrane potential, decrease mitochondrial activity (MitoTracker Orange staining), and up-regulate the expression of mitochondrial apoptosis-related proteins. Further examinations revealed that the expression level of FOXO3a and PUMA in NSCLC was significantly increased by genistein.

**Discussion and conclusions:**

Our data may provide basic information for further development of genistein as a promising lead compound targeting NSCLC by inducing mitochondrial apoptosis.

## Introduction

The World Health Organization (WHO) published the ‘World Cancer Report’ in 2020, revealing that lung carcinoma is the second most common type of cancer and a major cause of mortality worldwide (Sung et al. 2021). Non-small cell lung cancer (NSCLC) remains by far the most common malignancy of lung carcinoma, which has caused increasing numbers of cancer-related death in recent years (Ye et al. 2021). Treatment approaches for NSCLC include surgery, chemotherapy, radiotherapy, immunotherapy and molecular targeted therapy (Lemjabbar-Alaoui et al. 2015). Chemotherapy is one of the most common strategies for addressing NSCLC, especially for patients whose condition is non-surgical (Cheepsattayakorn and Cheepsattayakorn 2014; Visconti et al. 2017). Even though chemotherapy alone, or in combination with other strategies, has brought about remarkable improvements in the survival and prognosis of NSCLC patients, a growing incidence of drug resistance and tumour proliferation in lung carcinoma is limiting the overall outcomes of chemotherapy for NSCLC. Thus, there are growing needs for the innovation of effective drugs with lower toxicity for the treatment of NSCLC.

Plant-derived natural compounds have proven to be a reliable and consistent source for medicinal chemistry research against cancer (Maiuthed et al. 2018; Kumar and Jaitak 2019; Subramaniam et al. 2019). Genistein is a typical phytoestrogenic compound that belongs to a multifunctional natural isoflavonoid class of flavonoids. This natural compound has been suggested by numerous preclinical investigations to potentially affect various molecular targets and modulate various signalling pathways in different types of cancer cells, such as breast cancer and colon cancer. Genistein could induce cell cycle arrest and reactive oxygen species (ROS)-dependent signalling, reverse EMT and suppress cancer cell stemness (Spagnuolo et al. 2015; Tuli et al. 2019; Zhang et al. 2019; Kaushik et al. 2019; Imai-Sumida et al. 2020; Mukund 2020). These experimental investigations have proposed a potential therapeutic role of genistein in cancer treatment.

In this study, we demonstrated that genistein could induce ROS generation, mitochondrial dysfunction, FOXO3a/PUMA expression and, ultimately, apoptosis in non-small lung cancer cells. Our findings suggest that genistein has the potential to be further developed as a novel leading compound against NSCLCs.

## Materials and methods

### Reagents

Genistein (with purity >97%) was purchased from Shanghai Maclin Biochemical (Shanghai, China) and dissolved in 0.1% DMSO before use. A CCK-8 assay kit (Solarbio, Beijing, China), AnnexinV-FITC/PI apoptosis detection kit, and DAPI were purchased from BD Pharmingen (Franklin Lakes, NJ). 2′,7′-dichlorodihydrofluorescein diacetate (DCFH-DA) and dihydroethidium (DHE) probes were purchased from Beyotime Biotech (Nantong, China). B cell lymphoma-2 (Bcl-2, #244614), Bcl-2-associated x protein (Bax, #B323312), cytochrome C (#288539) and FOXO3a (#B217778) were purchased from Biolegend; GAPDH (#BM3896) was purchased from Boster Biological Technology; PUMA (#GR3239989-14) was purchased from Abcam; p-Akt (#DZE1819111) was purchased from Biotechne; and HPR-labeled goat anti-rabbit IgG (#A020) and HPR-labeled goat anti-mouse IgG (#8A0216) were purchased from the Beyotime Institute of Biotechnology (Nantong, China). MitoTracker Orange CMTMRos (M7510) was purchased from ThermoFisher Scientific (Waltham, MA).

### Cell lines

Human lung cancer A549 and 95D cell lines were purchased from the Institute of Biochemistry and Cell Biology, Chinese Academy of Sciences (Shanghai, China). The cells were cultured in DMEM (Gibco, Carlsbad, CA) supplemented with 10% heat-inactivated foetal bovine serum (Gibco, Darmstadt, Germany) and penicillin/streptomycin/gentamicin mixed solution (100× triple antibodic, Solarbio, Beijing, China) at 37 °C in a humidified CO_2_ incubator, and subcultured as required with trypsin/EDTA (0.25%).

### Cell viability assay

The cytotoxic effect of genistein against A549 and 95D lung cancer cells was detected by CCK8 assay. In brief, cells were seeded at a density of 4 × 10^3^ per well in 96-well plates and allowed to attach overnight before being treated with different concentrations of genistein, medium with same concentration of DMSO was used as a control. Cells were then cultured for another 48 h before 10 μL of CCK-8 reagent was added, and cultured for an additional 2 h. The plates were then measured at 450 nm using a full wavelength microplate reader (Thermo Scientific™ Multiskan GO).

### Colony formation assay

For colony formation assay, lung cancer A549 and 95D cells were seeded at 400 cells per well in a 6-well plate and cultured 24 h before treatment with genistein (0, 10, 20 and 40 μM), and were then cultured for 14 d. Th**e** clones were stained with 1% crystal violet solution (Solarbio, Beijing, China) for 15 min. After repeated washing with PBS until the crystal solution was washed away, the colonies were imaged using a camera, and colonies consisting of at least 50 cells were counted under a light microscope.

### Apoptosis assays

An Annexin V-FITC apoptosis kit from BD Pharmingen (Franklin Lakes, NJ) was used to detect apoptosis induced by genistein in A549 and 95D cells according to the manufacturer’s instructions. The 95D and A549 cells were plated at 1 × 10^5^ cells per well in 6-well culture plates for 12 h. Cells were then exposed to genistein with a final concentration of 0, 50, 100 and 150 μM for 48 h. After incubation, cells were collected, washed thrice with ice-cold PBS, and resuspended in 100 μL of binding buffer. Then, 5 μL of Annexin V-FITC and 5 μL PI were added and incubated in the dark at 37 °C for 20 min. After the described double staining, a total of 10,000 cells per sample were counted, and analysed with a FACS Calibur flow cytometer. Annexin V-FITC/PI double-stained cells were observed by using image flow cytometry (ImageStreamX MkII instrument, Amni, Luminex).

### DNA fragmentation detection by TUNEL assay

For the TUNEL assay, 95D and A549 cells were seeded at 1 × 10^5^ cells per well in 6-well plates. After overnight incubation, the cells were treated with genistein (0, 50, 100 and 150 μM) for 48 h. After incubation, the TUNEL detection assay (KeyGEN BioTECH, Nanjing, China) was performed according to the manufacturer’s instructions. A total of 10,000 cells per sample were analysed with a FACS Calibur flow cytometer. DAPI staining in combination with the TUNEL assay was also used for the morphologic observation of apoptotic cell death induced by genistein, by using image flow cytometry (ImageStreamX MkII instrument, Amni, Luminex).

### Reactive oxygen species (ROS) generation detection

Cellular ROS generation in A549 and 95D cells induced by genistein was measured by flow cytometry, utilizing a ROS detection kit (KeyGEN BioTECH, Nanjing, China). Briefly, 1 × 10^5^ cells were seeded in 6-well plates and cultured overnight before being cultured with 1 μL DCFH-DA at 37 °C. Cells were then treated with genistein (with a final concentration of 100 μM) for 0, 15, 30, 60 and 90 min. ROS levels were measured by FACS Calibur flow cytometry, and a total of 10,000 cells per sample were collected and analysed.

### Detection of mitochondrial membrane potential

We used JC-1(CBIC2(3), CAS: 3520-43-2) dye to monitor mitochondrial potential in 95D and A549 cells that had been changed by genistein. In brief, cells were seeded at 1 × 10^5^ cells per well in 6-well plates, and cultured overnight before treatment with genistein (0, 100 μM) for 24 h. Cells were collected and stained with JC-1 before being detected with a FACS Calibur flow cytometer. A total of 10,000 cells per sample were collected and analysed.

### MitoTracker orange staining assay

A549 and 95D cells were seeded in 6-well plates and cultured overnight before being exposed to genistein with final concentration of 0, 50, 100 and 150 μM, and were then cultured for 48 h. After incubation, cells were collected, washed thrice with ice-cold PBS, and resuspended in 400 μL of MitoTracker Orange staining solution (Thermo Fisher Scientific, CA) in the dark at 37 °C for 20 min. After the described staining, a total of 10,000 cells per sample were counted, and analysed with a FACS Calibur flow cytometer. Mitotracker Orange CMTMRos-stained cells were observed by using image flow cytometry (ImageStreamX MkII instrument, Amni, Luminex).

### Western blot

Western blotting was performed to analyse the expression of the proteins cytochrome C, Bax, Bcl-2, p-Akt, FoxO3a and PUMA in A549 and 95D cells. Briefly, cells treated with different concentrations of genistein were collected, and lysed in ice-cold RIPA lysis buffer with protease inhibitors for 30 min. Then, 20 μg of cell protein from each sample was separated using 12% SDS–PAGE and transferred onto a nitrocellulose membrane. The membrane was blocked for 1 h at room temperature using 5% non-fat milk in Tris-buffered saline containing 0.1% Tween-20 (TBST), before being incubated with primary antibodies overnight at 4 °C (dilution ratio 1:1000). After rinsing with TBST three times, the membranes were subsequently incubated with secondary antibodies for 1 h at room temperature. The targeted protein signal was visualized by incubation using an enhanced chemiluminescence system (ThermoFisher Scientific, OL191210A, MA).

### Immunofluorescence detected by imaging flow cytometry

Human lung cancer A549 and 95D cells were exposed to genistein (0, 50, 100 and 150 μM) for 48 h before harvest. Cells were then fixed for 15 min at room temperature with Fix I solution (Fluidigm, USA) buffer, and permeabilized with ice-cold methanol (80%) for 15 min. After triple washing with cell-staining buffer (BD, NJ), cells were incubated with FOXO3a (Biolegend, San Diego, CA, #B217778, 1:50) and PUMA (Abcam, Cambridge, UK, #GR3239989-14, 1:50) primary antibodies for 30 min, followed by incubation with anti-mouse PE (Biolegend, #401319, 1:50) or anti-rabbit FITC (Biolegend, #406403, 1:50) for 30 min. Cells were then detected using an ImageStreamX MkII instrument (Amni, Luminex), and analysed with IDEAS Software.

### Statistical analysis

All the experiment data are expressed as the mean ± SD. Comparisons among groups were assessed using Student’s *t*-test and one-way analysis of variance (ANOVA). Statistical significance was accepted when the *p* value was less than 0.05. Each experiment was conducted at least three times.

## Results

### Genistein administration decreased cell viability in human lung cancer cells

**Figure 1.**
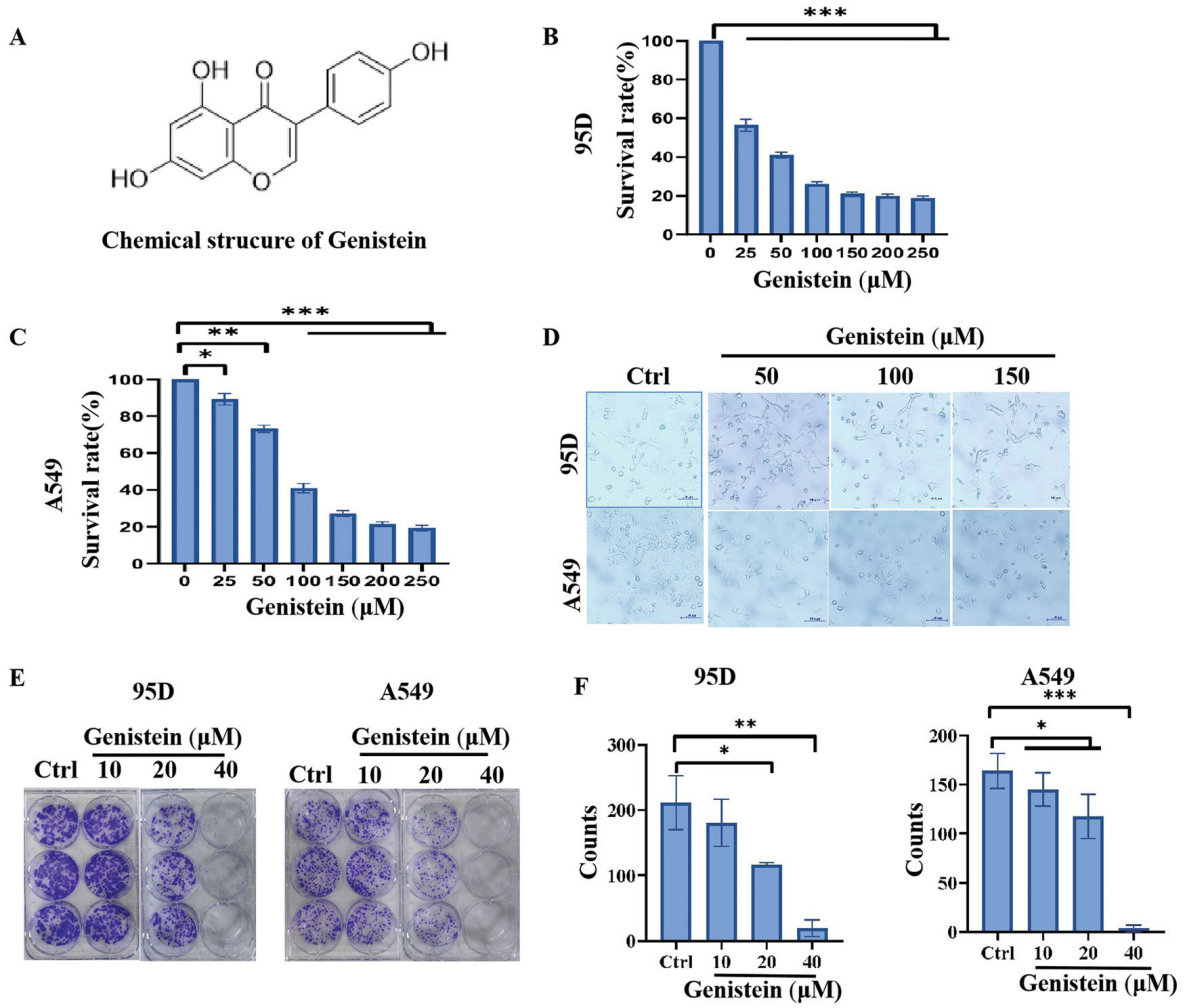
Effects of genistein on A549 and 95D lung cancer cells. (A) Chemical structure of genistein. (B, C) Cell viability of A459 and 95D lung cancer cells after administration of genistein for 48 h, as detected by CCK-8 assay. (D) Morphological changes in A459 and 95D lung cancer cells treated with different concentrations of genistein for 48 h. (E, F) Colony formation assay of A459 and 95D lung cancer cells treated with different concentrations of genistein (*p < 0.05, **p < 0.01, ***p < 0.001; *n* = 3 independent samples per group).

To determine the cytotoxic effect of genistein (the chemical structure of which is shown in Figure 1(A)) on the growth of 95D and A549 lung cancer cells, cell viability was assessed by the CCK8 assay, as described in previous publications. The results shown in Figure 1(B,C) indicate that genistein decreased lung cancer cell viability in a concentration-dependent manner (IC_50_ values were 32.96 ± 2.91 and 110.6 ± 2.41 μM for 95D cells and A549 cells, respectively). Morphological changes in A549 and 95D cells following treatment with different concentrations of genistein (0, 50, 100 and 150 μM) for 48 h were observed by light microscope, and the images of cells in different groups are shown in Figure 1(D). With an increased dose of genistein, the number of cells was decreased, and the cells revealed significant changes in cell morphology, including cellular shrinkage and rounding. Additionally, we observed that genistein exhibited the potential to inhibit the colony-forming ability of 95D and A549 lung cancer cells in a dose-dependent manner, as shown in Figure 1(E,F).

### Genistein-induced apoptosis in lung cancer A549 and 95D cells

**Figure 2.**
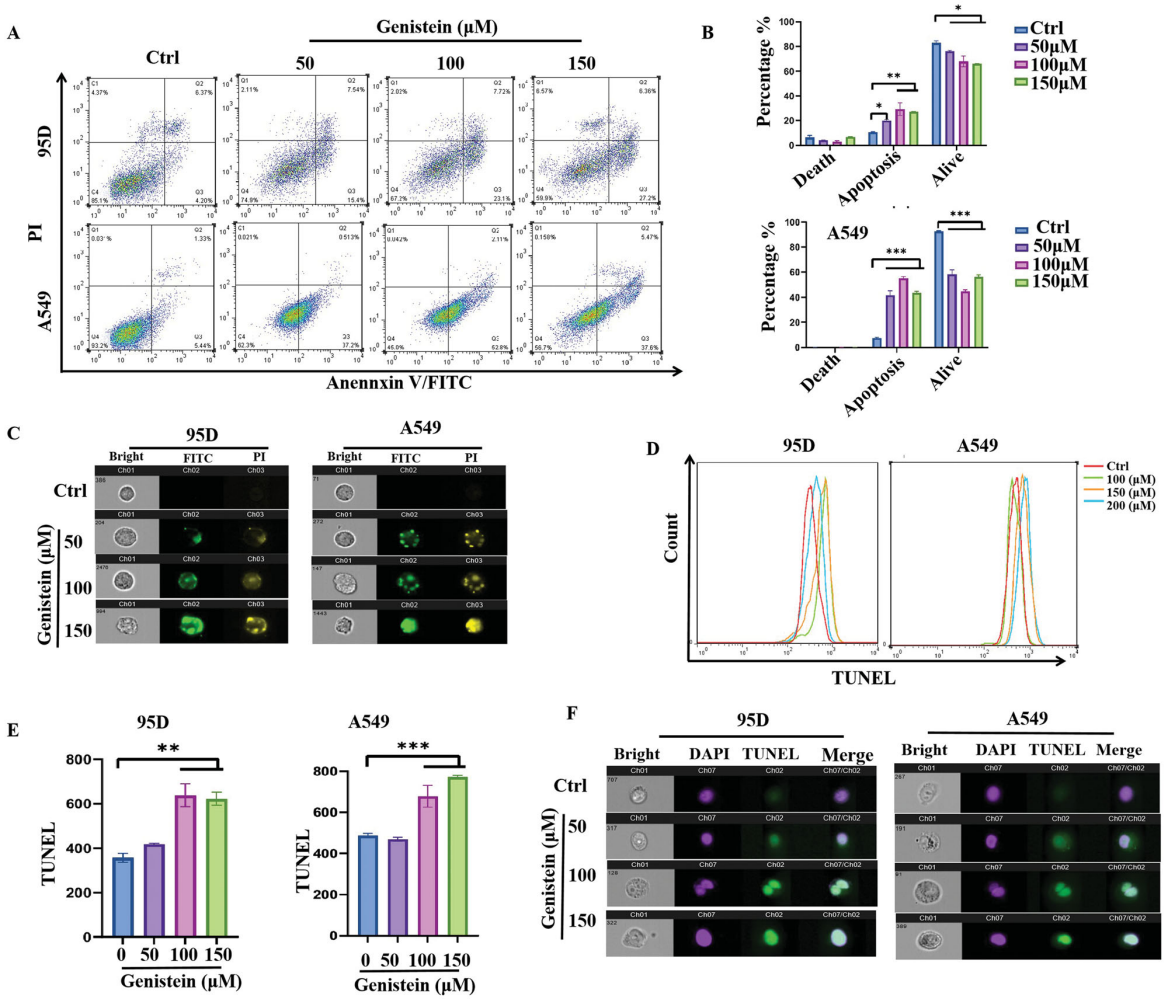
Genistein-induced apoptosis in A549 and 95D lung cancer cells. (A) Apoptosis induced by genistein was detected by flow cytometry using an Annexin V FITC/PI double-staining kit. (B) Statistical analysis of apoptotic death induced by genistein. (C) Images of apoptotic death induced by genistein in A549 and 95D cells, detected using imaging flow cytometry. (D) A549 and 95D cells were cultured with different concentrations of genistein for 48 h before a TUNEL assay was conducted, and were detected by flow cytometry. (E) Quantification of fluorescence intensity, as represented by TUNEL, induced by genistein. (F) Images of A549 and 95D cells treated with genistein for 48 h after being stained with a TUNEL probe and DAPI (*p< 0.05, **p< 0.01 and ***p< 0.001 when compared with control; *n* = 3 independent samples per group).

We used the AnnexinV-FITC/PI double-staining assay and TUNEL assay to evaluate the apoptosis induced in lung cancer cells by genistein. As shown in Figure 2(A,B), different concentrations of genistein were administered to A549 and 95D cells; the percentage of apoptotic death cells was 20.03%, 29.26% and 27.14% in 95D cells, and 41.62%, 55.24% and 43.45% in A549 cells when treated with 50, 100 and 150 μM genistein, respectively. Cell images after AnnexinV-FITC/PI double staining were also captured by imaging flow cytometry, as shown in Figure 2(C).

We then examined apoptosis in A549 and 95D cells using a TUNEL assay. Cells were treated with genistein (0, 50, 100 and 150 μM) for 48 h before being fixed and permeabilized, then incubated with rTdT buffer solution. The fluorescence intensity was detected by a BD Calibur flow cytometer, and the results are shown in Figure 2(D,E). The histogram plot (Figure 2(D)) illustrates that the density of apoptotic cells increased when exposed to genistein. Fluorescence images of A549 and 95D cells double stained with DAPI and TUNEL treated with different concentrations of genistein were captured using imaging flow cytometry, and are shown in Figure 2(F). As we can observe, the fluorescence intensity of DAPI and TUNEL was increased after treatment with genistein, indicating increased apoptotic death induced by genistein in A549 and 95D cells.

### Mitochondrial dysfunction induced by genistein in A549 and 95D cells

**Figure 3.**
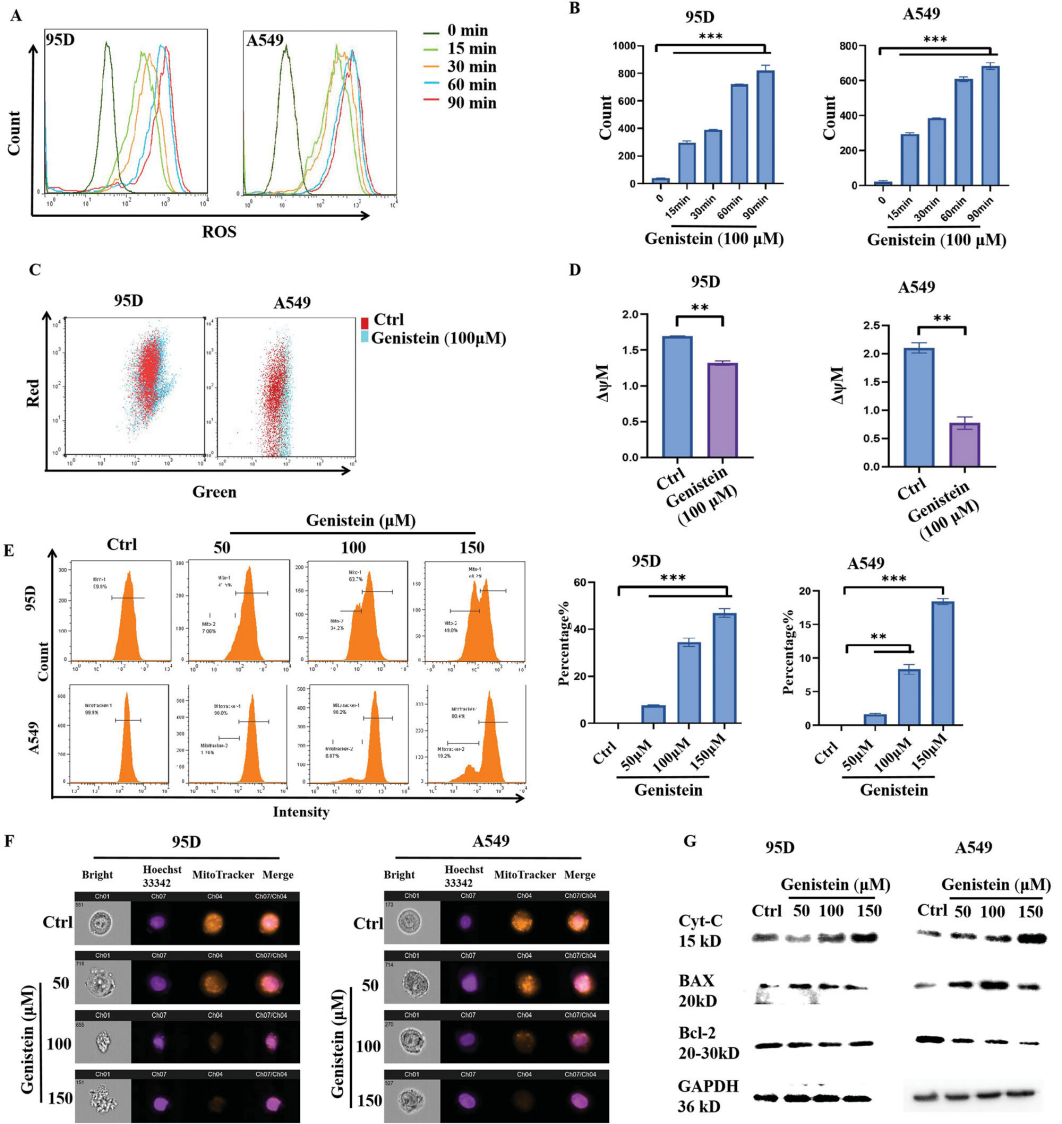
Effects of genistein-induced ROS generation and mitochondrial dysfunction in A549 and 95D cells. (A) Intracellular ROS generation was measured by DCFH-DA assay and detected by flow cytometry. (B) Quantification of ROS generation induced by genistein. (C) The effect of genistein on mitochondrial membrane potential detected by a flow cytometer using JC-1 assay. (D) Quantification of mitochondrial membrane potential in A549 and 95D cells that was changed by genistein. (E) Histograms of MitoTracker Orange fluorescence of A549 and 95D cells; percentage of cells with decreased fluorescence was analysed and is represented as the mean ± SD. (F) The mitochondria (MitoTracker Orange) and nucleus (Hoechst, blue) of A549 and 95D cells were detected by image flow cytometry. (G) The protein levels of Bcl-2 family members and cytochrome C expression were examined by Western blot analysis. Cells were collected after 48 h of culture with different concentrations of genistein (*p< 0.05, **p< 0.01 and ***p< 0.001 when compared with control; *n* = 3 independent samples per group).

Genistein is a phytochemical that has been reported to elevate intracellular ROS generation in cancer cells, such as melanoma and breast cancer cells (Jenie et al. 2019; Park et al. 2019). To explore whether genistein could induce ROS generation in lung cancer cells, a DCF-DA assay was performed. As we can see from Figure 3(A), ROS generation was dramatically induced in A549 and 95D cells when they were treated with 100 μM genistein for 15, 30, 60 and 90 min. According to previous publications, chemically induced abnormal ROS generation could lead to mitochondrial dysfunction in cancer cells. To monitor mitochondrial potential in 95D and A549 cells that had been changed by genistein, we then performed a JC-1 assay. As shown in Figure 3(C), the cytometry results revealed that genistein treatment could induce a decrease in mitochondrial membrane potential (ΔψM), ΔψM value was 2.1 ± 0.09, and 1.69 ± 0.01 in control group, and was 0.69 ± 0.05 and 1.31 ± 0.03 after genistein treatment, in A549 and 95D cells, respectively. We also used the MitoTracker Orange staining assay to evaluate the mitochondrial activity. As the results in Figure 3(E,F) show, MitoTracker activity was decreased by genistein in A549 and 95D cells in a concentration-dependent manner. Moreover, Western blot analysis was performed to evaluate the expression of mitochondrial apoptosis-related proteins such as cytochrome c, Bcl-2 and Bax. As Figure 3(G) shows, in A549 and 95D cells that had been exposed to genistein, levels of cytochrome c and Bax were significantly increased, while Bcl-2 was decreased, in a dose-dependent manner.

### Activation of FOXO3a/PUMA signalling by genistein in A549 and 95D cells

**Figure 4.**
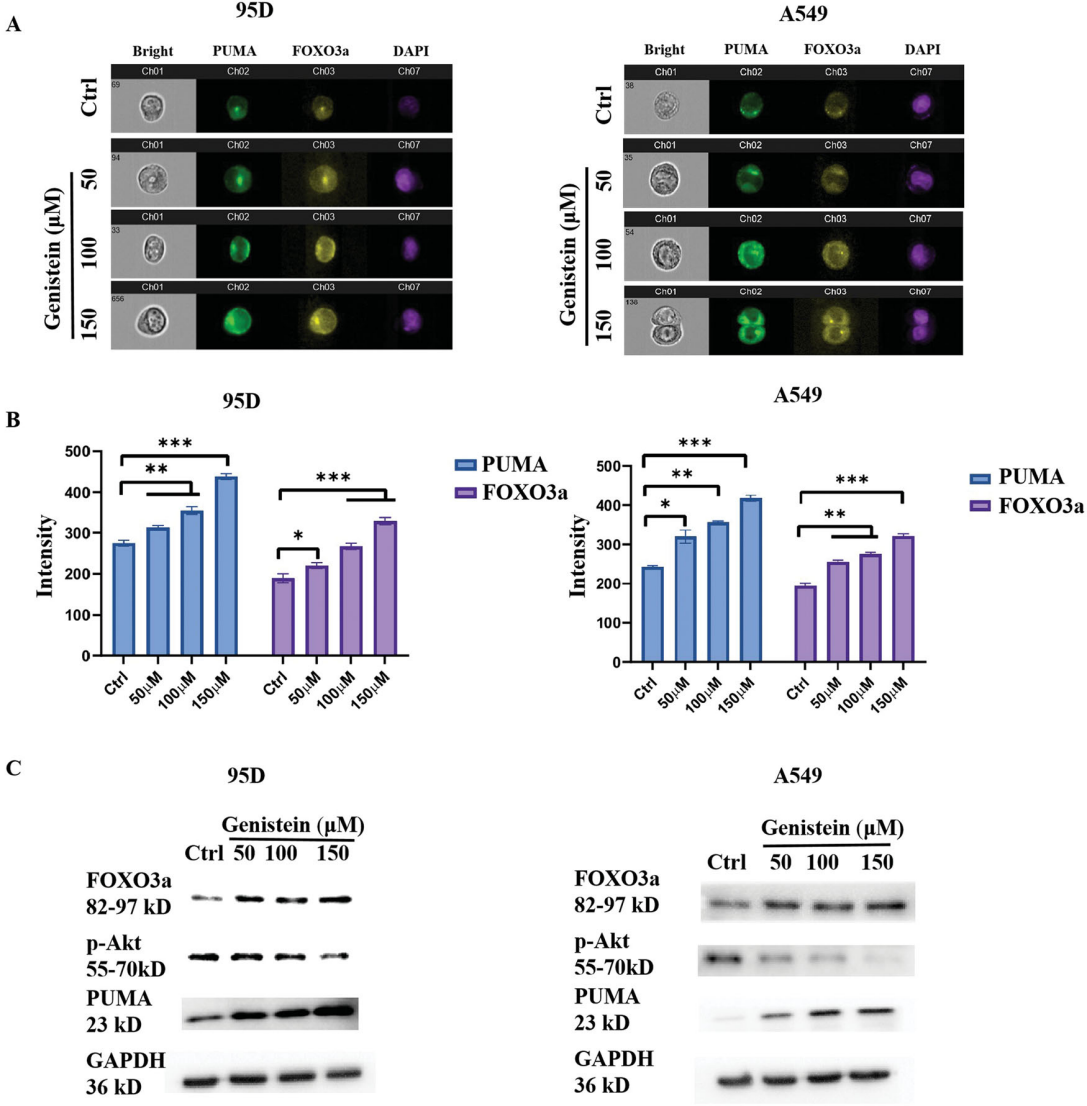
Genistein increased FOXO3a, and PUMA expression. (A) FOXO3a and PUMA expression was detected by imaging flow cytometry. (B) Quantification of PUMA and FOXO3a expression induced by genistein. (C) Protein expression levels of FOXO3a, p-Akt, and PUMA in A549 and 95 D cells treated with different concentrations of genistein were detected by Western blot analysis (*p < 0.05, **p < 0.01 and ***p < 0.001 when compared with control; *n* = 3 independent samples per group).

FOXO3a is a forkhead box transcription factor class O (FOXO) family protein, which was reported to be expressed in response to oxidative stress, and could activate multiple target genes including PUMA to induce apoptosis (Fasano et al. 2019; Zheng et al. 2020). In further analysis, we used imaging flow cytometry and Western blot assay to observe FOXO3a and PUMA expression induced by genistein in A549 and 95D cells. As shown in Figure 4(A,B), FoxO3a and PUMA expression was dramatically upregulated by genistein in A549 and 95D cells. Consistently, the results from the Western blot assay, as displayed in Figure 4(C), reveal that the protein expression levels of FOXO3a and PUMA were upregulated, but p-Akt was inhibited in A549 and 95D cells after treatment with different concentrations of genistein.

## Discussion

Malignant NSCLC possesses high-risk traits for promoting metastases. Despite recent advances in therapeutic strategies, cancer recurrence, metastasis and drug resistance are still challenges. Thus, there is a requirement for the development of more efficient and selective potential novel lead compounds against NSCLC. In recent years, much attention has been paid to natural agents possessing anticancer activity and with lower adverse effects. Genistein is included among these compounds, and has been shown to exhibit anticancer activities in numerous preclinical investigations, with non-cytotoxicity *in vivo* (Chae et al. 2019).

In this study, genistein was administered against the NSCLC cell lines A549 and 95D for its potential anticancer activity. Our results indicate that genistein exhibits significant anticancer activity against A549 and 95D cells in a dose-dependent manner. The IC_50_ of genistein against the A549 and 95D cells were 110.6 ± 2.41 and 32.96 ± 2.91 μM, respectively. The colony-forming capacity of A549 and 95D lung cancer cells was dramatically inhibited by genistein. In addition, we also observed that apoptotic cell death in A549 and 95D lung cancer cells was significantly induced by genistein detected using the AnnexinV and TUNEL assays. These results suggest that genistein is a potential anti-cancer agent for the treatment of NSCLC.

It is well established that genistein can induce ROS generation in cancer cells, and the overexpression of ROS could trigger mitochondrial dysfunction and induce apoptosis (Li et al. 2003). In our study, we discovered that intracellular ROS could be significantly induced by genistein in a time-dependent manner, and the same concentration of genistein could also decrease mitochondrial membrane potential (ΔψM). Thus, we proposed that the mitochondrial apoptotic pathway in NSCLC was involved in genistein-induced apoptotic cell death. As Bcl-2 family proteins are frequently main players in the apoptotic pathway of mitochondrial origin, we detected the changes in the expression levels of related proteins. The results show that cytochrome C release was induced by genistein, and genistein could also decrease Bcl-2 expression and increase Bax protein level. These results indicate that the mitochondrial apoptosis pathway was involved in genistein-induced cell death in NSCLC.

According to previous publications, over-expression of ROS could modulate forkhead O3A (FOXO3a) activation and cause cytotoxicity in cancer cells (Nasimian et al. 2020; Rajamanickam et al. 2020). FOXO3a transcriptionally regulates the expression of several target genes, such as BIM, NOXA and p53 upregulated modulator of apoptosis (PUMA), which promote apoptosis (Fasano et al. 2019). Based on this, we performed an examination of the FOXO3a and PUMA expression induced by genistein in A549 and 95D lung cancer cells. The results indicate that genistein could inhibit phosphorylation of AKT, and promote FOXO3a/PUMA expression.

## Conclusions

Our results suggest that genistein exhibits significant anticancer activity against non-small lung cancer A549 and 95D cells, indicating that genistein could be considered as a potential novel lead compound for the development of anti-lung cancer agents.
